# Occurrence and Genomic Characterization of ESBL-Producing, MCR-1-Harboring *Escherichia coli* in Farming Soil

**DOI:** 10.3389/fmicb.2017.02510

**Published:** 2017-12-14

**Authors:** Beiwen Zheng, Chen Huang, Hao Xu, Lihua Guo, Jing Zhang, Xin Wang, Xiawei Jiang, Xiao Yu, Linfeng Jin, Xuewen Li, Youjun Feng, Yonghong Xiao, Lanjuan Li

**Affiliations:** ^1^Collaborative Innovation Center for Diagnosis and Treatment of Infectious Diseases, State Key Laboratory for Diagnosis and Treatment of Infectious Diseases, The First Affiliated Hospital of College of Medicine, Zhejiang University, Hangzhou, China; ^2^Department of Respiratory Diseases, The First Affiliated Hospital of College of Medicine, Zhejiang University, Hangzhou, China; ^3^School of Laboratory Medicine and Life Science, Wenzhou Medical University, Wenzhou, China; ^4^College of Basic Medical Sciences, Zhejiang Chinese Medical University, Hangzhou, China; ^5^School of Public Health, Shandong University, Jinan, China; ^6^Department of Medical Microbiology and Parasitology, Zhejiang University School of Medicine, Hangzhou, China

**Keywords:** *mcr-1*, ESBLs, *Escherichia coli*, farming soil, animal manure

## Abstract

The emergence and spread of the mobile colistin resistance gene (*mcr-1*) has become a major global public health concern. So far, this gene has been widely detected in food animals, pets, food, and humans. However, there is little information on the contamination of *mcr-1*-containing bacteria in farming soils. In August 2016, a survey of ESBL-producing *Escherichia coli* isolated from farming soils was conducted in Shandong Province, China. We observed colistin resistance in 12 of 53 (22.6%) ESBL-producing Enterobacteriaceae isolates from farming soil. Six *mcr-1*-positive *E. coli* strains originating from a livestock-intensive area were found. The isolates belonged to four different STs (ST2060, ST3014, ST6756, and ST1560) and harbored extensive additional resistance genes. An *E. coli* with *bla*_NDM-1_ was also detected in a soil sample from the same area. Comparative whole genome sequencing and S1-PFGE analysis indicated that *mcr-1* was chromosomally encoded in four isolates and located on IncHI2 plasmids in two isolates. To our knowledge, we report the first isolation of *mcr-1* in ESBL-producing *E. coli* from farming soils. This work highlights the importance of active surveillance of colistin-resistant organisms in soil. Moreover, investigations addressing the influence of animal manure application on the transmission of *mcr-1-*producing bacteria are also warranted.

## Introduction

Antimicrobial resistance determinants, the dissemination of which are facilitated by human activities, are increasingly being recognized as emerging environmental contaminants with the potential to pose a threat to human health (Sanderson et al., 2016). It is well-recognized that large amounts of antibiotics are released from humans and animals into agricultural fields by manure fertilization (Jechalke et al., 2014). Subsequently, these substances may affect the structure and function of *in situ* bacterial communities and further lead to an increased abundance and transferability of antibiotic resistance genes (ARGs) (Jechalke et al., 2014). Extended-spectrum β-lactamase (ESBL)-producing Enterobacteriaceae is an important group of multidrug-resistant (MDR) bacteria which constitutes a major public health concern (Bush and Fisher, 2011). Antimicrobial therapy with colistin alone, or in combination with other antibiotics, is regarded as a “last-line” treatment option against bacterial infections caused by MDR Gram-negative pathogens (Paterson and Harris, 2016). Globally, there are increasing reports of colistin-resistant Enterobacteriaceae. Bacteria that produce ESBLs or carbapenemases in particular, are associated with colistin resistance; these colistin-resistant bacteria pose a severe health threat due to the limited therapeutic options available (van Duin and Doi, 2015).

Recently, concerns were raised regarding the increasing prevalence of colistin-resistant Enterobacteriaceae due to the discovery of the first plasmid-mediated colistin resistance gene, *mcr-1*, which was identified in China (Liu et al., 2016). Since the first report of *mcr-1*, *mcr* genes, including *mcr-1/2/3/4/5* have been detected in animals, food, human microbiota, and clinical samples in over 30 countries (Gao et al., 2016; Xavier et al., 2016; Borowiak et al., 2017; Carattoli et al., 2017; Yin et al., 2017). Notably, our and other research groups have already found Enterobacteriaceae isolates containing MCR-1 and carbapenemases, raising serious concerns about the possible global dissemination and spread of pan-resistant pathogens (Zheng et al., 2016).

To date, the *mcr* gene has been detected worldwide in human and animal samples; however, its occurrence in environmental samples has rarely been described. Several previous studies have documented the emergence of *mcr*-harboring, ESBL-producing Enterobacteriaceae in river and waste water (Zurfuh et al., 2016; Ovejero et al., 2017; Sun P. et al., 2017), suggesting that the *mcr* gene has spread from veterinary to aquatic environments. Colistin resistance is a threat to human and animal health worldwide, and soil ecosystems are one of the major environmental contamination sectors of antibiotic-resistant bacteria. However, the extent and significance of emergence of MCR-producing isolates in soil has not been elucidated.

The aim of this study was to describe the occurrence of *Escherichia coli* isolates harboring both the *bla*_CTX-M_ and *mcr* genes that were originally isolated from farming soils in China. We also sought to reveal the genomic structure of *mcr*-positive *E. coli* isolates and to decipher the colistin resistance mechanisms among these environmental isolates.

## Materials and Methods

### Study Site and Soil Sampling

In August 2016, we collected farming soil samples from 32 distinct rural sites in Shandong Province, China (Supplementary Figure S1). The families at the study sites most commonly lived in a four-room house with an outdoor toilet located in the yard. Most families kept chicken and pigs in the yard. Toilet waste was disposed by the family itself and manure from animals were often applied to agricultural fields. Three non-repeated samples were obtained from each site, which is geo-positioned with a precision <0.5 m. All samples were collected from deeper layers (depth 3–10 cm) within a 20 cm × 20 cm area and kept on ice during transport.

### Isolation of ESBL-Producers

Each sample (2.0 g) was homogenized with a fivefold volume of sterile Luria-Bertani (LB) liquid medium (∼10 ml) and cultured at 37°C overnight. The enriched solutions were plated on MacConkey agar plates with 2 mg/L cefotaxime for 18–24 h at 37°C to isolate potential ESBL-producing strains. ESBL production was confirmed via the double-disk synergy test (DDST) in accordance with Clinical and Laboratory Standards Institute (CLSI) guidelines (CLSI, 2017). ESBL-producing isolates were identified by matrix-assisted laser desorption ionization-time of flight mass spectrometry (MALDI-TOF MS).

### Antimicrobial Susceptibility Testing and Detection of Resistance Genes

Broth microdilution was performed for antimicrobial susceptibility testing of ESBL producers, and the results were interpreted using CLSI breakpoints. EUCAST breakpoints were used for colistin and tigecycline^1^. The ESBL-producing isolates were further subjected to PCR for the detection of *mcr* genes (*mcr-1*, *mcr-2*, *mcr-3*, and *mcr-4*), carbapenemase genes and ESBL genes, as previously described (Branas et al., 2015; Liu et al., 2016; Xavier et al., 2016; Carattoli et al., 2017; Yin et al., 2017).

### Multilocus Sequence Typing and Pulsed-Field Gel Electrophoresis

Multilocus sequence typing (MLST) was undertaken in accordance with protocols described in the *E. coli* database (Wirth et al., 2006) and the *Klebsiella pneumoniae* database (Brisse et al., 2009). The clonality of *mcr-1*-positive isolates was assessed by XbaI-pulsed-field gel electrophoresis (PFGE) and cutoff lines at 85% were used to analyze genetic relatedness (Zheng et al., 2015). S1-PFGE, hybridization, and conjugation experiments were performed as previously described (Zheng et al., 2016).

### Whole Genome Sequencing (WGS) and *in Silico* Analyses

To characterize the genetic features of the *mcr*-bearing isolates, whole-genome sequencing (WGS) was performed on six isolates using the Illumina HiSeq platform (Illumina, San Diego, CA, United States). WGS data quality control was performed as previously described (Zhang et al., 2014). Sequencing data were assembled using SOAPdenovo (Luo et al., 2012) and queries were then generated by utilizing the ResFinder 2.1 (Zankari et al., 2012) database to identify acquired ARGs. PlasmidFinder 1.3 was employed to identify plasmid replicon types (Carattoli et al., 2014). Plasmid profiling using plasmidSPAdes to assemble plasmids from WGS data was also performed (Antipov et al., 2016).

### Conjugation Experiments and Plasmid Analysis

The transferability of *mcr*-bearing plasmids from isolates was determined using filter mating with *E. coli* J53 as the recipient strain, mixed at a ratio of 1:1 in broth culture, as previously described (Zheng et al., 2015). The resulting transconjugants were selected on BHI agar plates amended with colistin (2 mg/L). The colonies were identified as *E. coli* J53 via MALDI-TOF MS and such colonies were screened and sequenced for the presence of *mcr-1* gene. Plasmid sizes were determined using the S1-nuclease PFGE (S1-PFGE) method (Zheng et al., 2015). Additionally, Southern blotting analysis was performed to determine genetic location using specific probes for the *mcr* gene. Identification of replicon types of the plasmid incompatibility (Inc) groups was performed by multiplex PCR, as described previously (Carattoli et al., 2005).

### Accession Numbers

The whole genome sequences of *mcr-1*-positive *E. coli* strains were deposited in GenBank under the following accession numbers: accession no. MVOR00000000 (E4), MVOS00000000 (E11), MVOT00000000 (E24), MVOU00000000 (E38), MVOV00000000 (E43), and MVOW00000000 (E47).

## Results and Discussion

### Identification of ESBL-Producing Enterobacteriaceae

Analysis of 96 soil samples led to the isolation of 53 ESBL-producing Enterobacteriaceae, including 42 *E. coli* isolates and 11 *K. pneumoniae* isolates. MIC results demonstrated that 50 (96.2%) isolates exhibited multidrug resistance, which was defined as resistance to at least three different classes of antimicrobial agents (Supplementary Table S1). The highest susceptibility rate was observed for imipenem (100%), followed by meropenem (96.2%), tigecycline (94.3%), colistin (79.2%), and polymyxin B (75.5%). *bla*_CTX-M_ genes were detected in 50 (96.2%) isolates. The most prevalent *bla*_CTX-M_ gene was *bla*_CTX-M-14_ (*n* = 21), followed by *bla*_CTX-M-27_ (*n* = 13), *bla*_CTX-M-65_ (*n* = 10), *bla*_CTX-M-55_ (*n* = 9), *bla*_CTX-M-11_ (*n* = 2), and *bla*_CTX-M-3_, *bla*_CTX-M-15_, and *bla*_CTX-M-17_ (*n* = 1 for each) (Supplementary Table S2). For *E. coli* in a clinical context, ST10, ST38, ST131, and ST405 are responsible for the dissemination of CTX-M worldwide (Hernandez and Gonzalez-Acuna, 2016). The STs among the ESBL-producing *E. coli* observed in this study were quite different and only ST10 (*n* = 2) was detected among the aforementioned STs. Notably, although NDM-1-producing strains are rarely recovered from soil (Wang and Sun, 2015), the *bla*_NDM-1_ gene was identified in strain E28 (Supplementary Table S1). In addition, 10 (23.8%) *E. coli* and 2 (18.2%) *K. pneumoniae* were resistant to colistin and polymyxin B. The currently known resistance mechanisms to colistin involve modifications of the lipopolysaccharide and can either be encoded chromosomally or by the plasmid-borne *mcr-1/2/3/4* (Poirel et al., 2017). In our study, six isolates were positive for *mcr-1* and none of the isolates carried *mcr-2/3/4* determinants. DNA sequencing of the full-length *mcr* gene revealed 100% matching nucleotide identity with the *mcr-1* sequence described in the original publication. Interestingly, *mcr-1*-producing isolates were recovered from five sampling sites, all of which were located in an area with intensive livestock farming (Supplementary Figure S1). In addition, except for isolates E31 and E7, isolates E91, E95, K63, and K64 were highly resistant to colistin (>16 mg/l). The resistance mechanism responsible for the high MICs observed could be due to mutations in the two-component system *pmrAB*, which can lead to increases in the extent of LPS modifications which in turn lowers the affinity to colistin (Poirel et al., 2017).

### Occurrence of MCR-1-Harboring *E. coli* in Farming Soil

**FIGURE 1:**
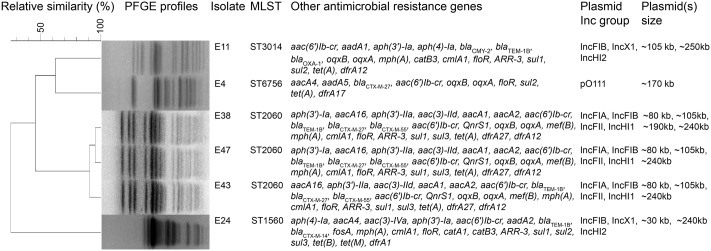
Molecular and genotypic profiles of six *mcr-1*-producing *Escherichia coli* isolates from farming soil. Summary of molecular epidemiological characteristics of the six *mcr-1*-producing *E. coli* isolates. The dendrogram of PFGE patterns was constructed using BioNumerics v6.6 with UPGMA clustering. The scale bar indicates percentage of genetic relatedness.

**FIGURE 2:**
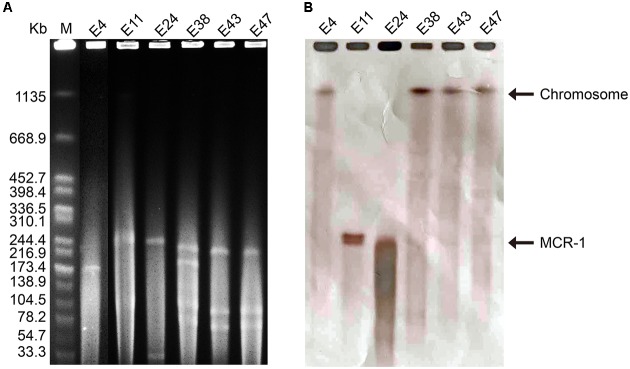
**(A)** Plasmid profiles of six *mcr-1*-positive isolates generated using the restriction enzyme S1, with *Salmonella enterica* serovar Branderup as the molecular mass marker. **(B)** Southern blot hybridization with a *mcr-1*-specific probe. The MCR-F1 (5′-TGCAGCATACTTCTGTGTGGT-3′) and MCR-R1 (5′-CACCGAGTAGATTGGCATGA-3′) primers were used.

The six *mcr-1*-producers belonged to ST2060 (*n* = 3), ST3014, ST6756, and ST1560 (**Figure 1**). These STs have not been previously reported to be associated with *mcr-1*. The diverse STs exhibited genetic heterogeneity, which has also been observed in other reports on MCR-1-producing *E. coli* (Veldman et al., 2016; Wang et al., 2016). These findings imply the complex genetic diversity of both the *mcr-1* gene and its *E. coli* hosts in soils in China. As a consequence, there is an urgent need to formulate a comprehensive strategy to prevent further dissemination of *mcr-1* in multidrug-resistant isolates. The isolates E38, E43, and E47 presented highly similar profiles, indicating the clonality of these MCR-1-producing strains (**Figure 1**). S1-PFGE and hybridization showed that the MCR-1-producing isolates had multiple plasmids that ranged from 30 to 250 kb (**Figure 2A**). Moreover, the *mcr-1* gene was located on a 220 kb plasmid in isolates E11 and E24. Interestingly, southern blot and conjugation experiments produced negative results for E4, E38, E43 and E47, indicating that the *mcr-1* gene was chromosomally encoded in these isolates (**Figure 2B** and Supplementary Table S3). Chromosome-based *mcr-1* genes have also been found in previous studies (Falgenhauer et al., 2016; Li et al., 2016). Our study revealed unexpected diversity in the *mcr-1*-harboring strains present in the examined soil samples.

China produces an estimated 2.1 trillion kg of swine and chicken annually (Zhou et al., 2016). Prior to the Chinese government’s ban of colistin as a feed additive for animals in Nov 1, 2016, the consumption of colistin was more than 8,000 tons (Walsh and Wu, 2016). The long-term usage of huge amounts of colistin may have established a selection pressure facilitating the generation and dissemination of colistin-resistant isolates in feces, especially in chicken, as antimicrobial agents were often administered orally to these animals (Nguyen et al., 2016). Predictably, colistin-resistant strains have been widely detected in fecal samples from food animals in China (Bai et al., 2016). To the best of our knowledge, no report to date have described *mcr*-positive Enterobacteriaceae isolated from soil samples. However, *mcr*-positive *E. coli* have been identified in river water, vegetable samples (Zurfuh et al., 2016) and sewage water (Ovejero et al., 2017). Interestingly, one study investigated the transmission of *mcr-1*-containing bacteria into the environment around farm areas in Germany and found seven *mcr-1*-positive *E. coli* strains originating from environmental boot swabs, dog feces, stable flies, and manure (Guenther et al., 2017). More pertinently, a recent report revealed that *mcr-1* producers have been identified in drinking water from Shandong Province (Sun P. et al., 2017). Notably, in rural areas of China, especially areas with intensive livestock farming, animal manure is widely used as organic fertilizer (Zhu et al., 2013). These findings were consistent with our results, although the contribution of soil-contaminant routes to the spread of *mcr-1*-harboring bacteria requires additional investigation. Our data suggest potential contamination of soil with bacteria harboring the *mcr-1* gene from animal manure, since in our study, all of the isolated *mcr-1-*producers were recovered from a livestock-intensive area.

### Genomics Features of MCR-1-Producing Isolates

Whole-genome sequencing produced 4,717,954, 5,886,228, 4,302,436, 5,043,375, 4,164,486, and 5,989,082 pairs of 150-bp reads for E4, E11, E24, E38, E43, and E47, respectively. Assembly of these isolates’ genomes resulted in 109, 179, 124, 116, 119 and 113 contigs larger than 500 bp, comprising 4.9 megabases of sequence and representing a median 309-fold coverage (Supplementary Table S4).

The wide-spread use of antibiotics in animal production leads to a contamination of animal feces and urine with the parent antimicrobial compound and MDR bacteria, resulting in contamination of the farming soils with ARGs (Xu et al., 2015). All of the sequenced *mcr-1*-positive isolates found in this study harbored multiple resistance genes, inducing multidrug resistance, and multiple plasmid Inc types, suggesting that multiple plasmids were present, a finding consistent with our plasmid profiling results (**Figure 2**). The *bla*_TEM-1B_, *floR*, and *sul1* genes and aminoglycoside resistance genes [*aac(6′)Ib-cr*, *aph(3′)-Ia* or *aadA*] were detected in all *mcr-1*-positive *E. coli* strains; these findings explain the extensively drug-resistant phenotype of these *E. coli* isolates (**Figure 1** and Supplementary Table S1). The E38, E43, and E47 strains were genetically closely related; this finding was consistent with our observations for PFGE profiles, indicating the isolate-driven spread of the *mcr-1* gene. Interestingly, isolates E11 and E24 shared the same plasmid Inc types although PFGE results showed their relative heterogeneity, indicating the prevalence of *mcr-1*-bearing plasmids in this livestock-intensive area and their broad-host-range characteristics which facilitates the dissemination of the *mcr-1* gene (**Figures 1**, **2**). A recent study also revealed that the worldwide dissemination of *mcr-1* was mainly mediated by highly promiscuous plasmids rather than several populations of *mcr-1*-carrying clones (Matamoros et al., 2017). The clones may have the intrinsic ability of acquiring antimicrobial resistance genes, including *mcr-1*, enabling them to play a potential role as a reservoir for this gene and facilitate the prevalence of *mcr-1* gene in local regions.

We identified plasmid replicons in all six isolates, including one type of plasmid in E4, three types of plasmids in E11, and four types of plasmids in E24, E38, E43, and E47. Via BLAST analysis of the plasmid sequences assembled by plasmidSPAdes, we also found seven different types of plasmids in these strains, a result consistent with the S1-PFGE findings (**Figure 2A**). In isolate E11, *mcr-1* was carried on an IncHI2 plasmid. A search of the nr/nt database revealed sequence homology between the assembled large plasmid contig (60.4 kb) and the annotated *mcr-1*-positive IncHI2 plasmid pHNSHP45-2 (GenBank: KU341381) (Supplementary Figure S2A). For isolate E24, a *mcr-1-*harboring contig (37.5 kb) was found to be 99% identical to the *mcr-1*-positive IncHI2 plasmid pMR0516mcr (GenBank: KX276657) (Supplementary Figure S2B). Notably, the sequence of *pap2*-*mcr-1*-IS*Apl1* region was identified in both plasmids, which is usually found in *mcr-1*-carrying plasmids (Wang et al., 2017). In addition, the genetic context of the chromosomally encoded *mcr-1* genes was similar to that reported in a previous study, i.e., *mcr-1* was observed in a structure consisting of IS*Apl1*-IRR-*mcr-1-hp* (Supplementary Figure S3) (Sun J. et al., 2017). IS*Apl1* is a member of the IS*30* family, and contributes to the mobilization of the *mcr-1* cassette into the chromosome through recognition of different related IRRs, which could perfectly match with 3′-end of *mcr-1*-*hp* to form a circular intermediate (Dona et al., 2017; Sun J. et al., 2017).

## Conclusion

To the best of our knowledge, this investigation involved the first survey of MCR-1 in ESBL-producing *E. coli* isolates from farming soils. It is well-known that the *mcr-1* gene can spread through food chains. This study further highlights the possibility that *mcr-1* may enter humans via soil contamination and thereby threaten public health. Rates of *mcr-1* carriage are likely to rise rapidly in the examined region due to the environmental contamination with *mcr-1* described in this work and a previous study (Sun P. et al., 2017). Therefore, investigations addressing the influence of animal manure application on the transmission of *mcr-1* producers are of great significance, and improved multisectoral surveillance for colistin-resistant *E. coli* in Zhucheng City and nearby regions is warranted.

## Author Contributions

BZ, YX, and LL conceived and designed the experiments. BZ, CH, HX, JZ, LJ, and XW performed the experiments. LG, XJ, and XY analyzed the data. BZ, XL, YF, and YX wrote the paper.

## Conflict of Interest Statement

The authors declare that the research was conducted in the absence of any commercial or financial relationships that could be construed as a potential conflict of interest.

## Footnotes

1 http://www.eucast.org
