# Supplementary material for: Occurrence and Genomic Characterization of ESBL-Producing, MCR-1-Harboring Escherichia coli in Farming Soil
Source: Front Microbiol. 2017 Dec 14;8:2510. doi: 10.3389/fmicb.2017.02510 (PMC5735249; doi:10.3389/fmicb.2017.02510)
Supplement: Supplementary file 1 [file Table_1.DOCX]

**SUPPORTING INFORMATION**

**Occurrence and genomic characterization of ESBL-producing, MCR-1-harboring *Escherichia coli* in farming soil**

*Running Title:* MCR-1-positive *Escherichia coli* in soil

Beiwen Zheng^a,1^, Chen Huang ^a,1^, Hao Xu ^a,1^, Lihua Guo^a^, Jing Zhang^a,b^, Xin Wang^a,c^, Xiawei Jiang^d^, Xuewen Li^e^, Youjun Feng^f^, Yonghong Xiao^a,^* and Lanjuan Li^a^

^a^ Collaborative Innovation Center for Diagnosis and Treatment of Infectious Diseases, State Key Laboratory for Diagnosis and Treatment of Infectious Diseases, The First Affiliated Hospital, College of Medicine, Zhejiang University, Hangzhou, China

^b^ Department of Respiratory Diseases, The First Affiliated Hospital, College of Medicine, Zhejiang University, Hangzhou, China

^c^ College of Basic Medical Sciences, Zhejiang Chinese Medical University, Hangzhou, China

^d^ College of Basic Medical Sciences, Zhejiang Chinese Medical University, Hangzhou, China

^e^ School of Public Health, Shandong University, Jinan, China

^f^ Department of Medical Microbiology and Parasitology, Zhejiang University School of Medicine, Hangzhou, China

^1^ Contributed equally to the work.

*Corresponding author. Tel: 86-571-87236421; Fax: 86-571-87236421; E-mail: [xiao-yonghong@163.com](mailto:xiao-yonghong@163.com)

**Table S1.** Antimicrobial drug resistance profiles of of ESBL-producing Enterobacteriaceae isolated from farming soils in China*

| IsolateID | Species |  | MIC for antimicrobial drugs tested, μg/mL† | | | | | | | | | | | |
| --- | --- | --- | --- | --- | --- | --- | --- | --- | --- | --- | --- | --- | --- | --- |
|  |  | AMK | CFZ | CIP | CST | CXM | FOX | GEN | IMP | MEM | PMB | PTZ | TET | TGC |
| E4 | *E. coli* | 8 | >128 | 64 | 8 | >128 | 8 | 64 | 0.125 | <0.06 | 8 | 4 | 128 | 0.25 |
| E11 | *E. coli* | 4 | >128 | 64 | 16 | >128 | 8 | 128 | 0.125 | <0.06 | 16 | 4 | 64 | 0.5 |
| E13 | *E. coli* | 2 | >128 | 4 | 0.125 | >128 | 8 | 064 | 0.5 | 0.125 | 0.5 | 32 | >128 | 2 |
| E14 | *E. coli* | 2 | >128 | 4 | 0.125 | >128 | 8 | 32 | 0.5 | <0.06 | 0.5 | 8 | >128 | 0.5 |
| E24 | *E. coli* | 2 | >128 | 2 | 4 | >128 | 4 | 64 | 0.125 | <0.06 | 4 | 2 | 128 | 0.25 |
| E25 | *E. coli* | 4 | >128 | 4 | 0.125 | >128 | 8 | 64 | 0.125 | <0.06 | 0.5 | 16 | 128 | 0.5 |
| E26 | *E. coli* | 2 | >128 | 42 | 0.25 | >128 | 4 | 64 | 0.125 | <0.06 | 0.5 | 4 | 128 | 0.5 |
| E28 | *E. coli* | 4 | >128 | 4 | 0.25 | >128 | >128 | 128 | 2 | 4 | 1 | 42 | 128 | 2 |
| E31 | *E. coli* | 16 | >128 | 64 | 8 | >128 | 4 | 64 | 0.125 | <0.06 | 8 | 4 | 128 | 0.5 |
| E38 | *E. coli* | 2 | >128 | 64 | 4 | >128 | 8 | 2 | <0.06 | <0.06 | 4 | 4 | 128 | 0.25 |
| E43 | *E. coli* | 4 | >128 | 2 | 4 | >128 | 16 | 64 | <0.06 | <0.06 | 2 | 4 | 128 | 0.5 |
| E45 | *E. coli* | 4 | >128 | 4 | 0.125 | >128 | 4 | 32 | 0.125 | <0.06 | 0.5 | 2 | 64 | 0.5 |
| E46 | *E. coli* | 4 | >128 | 0.5 | 0.125 | >128 | 4 | 4 | 0.125 | <0.06 | 0.5 | 2 | 64 | 0.5 |
| E47 | *E. coli* | 4 | >128 | >128 | 4 | >128 | 8 | 128 | 0.125 | <0.06 | 4 | 4 | 128 | 0.5 |
| E51 | *E. coli* | 2 | >128 | 1 | 0.25 | >128 | 4 | 2 | 0.125 | <0.06 | 0.5 | 4 | 64 | 0.125 |
| E52 | *E. coli* | 4 | >128 | 16 | 0.125 | >128 | 2 | 8 | <0.06 | <0.06 | 0.5 | 4 | 64 | 0.125 |
| E53 | *E. coli* | 4 | >128 | 32 | 0.125 | >128 | 8 | 128 | <0.06 | <0.06 | 0.5 | 2 | 128 | 0.5 |
| E54 | *E. coli* | 4 | >128 | 0.5 | 0.25 | >128 | 4 | 128 | 0.125 | <0.06 | 0.5 | 8 | 128 | 0.5 |
| E55 | *E. coli* | 4 | >128 | 8 | 0.125 | >128 | 4 | 64 | <0.06 | <0.06 | 0.5 | 2 | 64 | 0.5 |
| E56 | *E. coli* | 4 | >128 | 8 | 0.125 | >128 | 4 | 64 | <0.06 | <0.06 | 0.5 | 2 | 64 | 0.5 |
| E57 | *E. coli* | 4 | >128 | 0.5 | 0.25 | >128 | 4 | 64 | 0.25 | <0.06 | 0.5 | 4 | 64 | 1 |
| E58 | *E. coli* | 4 | >128 | 0.5 | 0.125 | >128 | 4 | 4 | 0.125 | <0.06 | 0.5 | 2 | 64 | 0.5 |
| E60 | *E. coli* | 4 | >128 | 0.03 | 0.25 | >128 | 64 | 4 | 0.25 | 0.25 | 0.5 | 4 | 64 | 0.125 |
| E70 | *E. coli* | 2 | >128 | >128 | 8 | >128 | 64 | 64 | 0.125 | <0.06 | 8 | 8 | 128 | 0.25 |
| E71 | *E. coli* | 2 | >128 | 32 | 0.5 | >128 | >128 | 2 | 1 | 4 | 1 | 32 | 64 | 2 |
| E73 | *E. coli* | 2 | >128 | 32 | 0.25 | >128 | 8 | 2 | 0.125 | <0.06 | 0.5 | 4 | 64 | 1 |
| E74 | *E. coli* | 2 | >128 | 16 | 0.25 | >128 | 8 | 2 | 0.125 | <0.06 | 0.5 | 4 | 64 | 1 |
| E75 | *E. coli* | 4 | >128 | 2 | 0.125 | >128 | 4 | 2 | <0.06 | <0.06 | 0.25 | 2 | 32 | 0.5 |
| E76 | *E. coli* | 4 | >128 | 2 | 0.125 | >128 | 4 | 4 | 0.125 | <0.06 | 0.5 | 4 | 32 | 0.5 |
| E78 | *E. coli* | 2 | >128 | 0.5 | 0.25 | >128 | 4 | 4 | 0.125 | <0.06 | 0.5 | 2 | 32 | 0.5 |
| E79 | *E. coli* | 8 | >128 | >128 | 0.125 | >128 | 8 | >128 | 0.125 | <0.06 | 0.5 | 8 | >128 | 0.25 |
| E80 | *E. coli* | 8 | >128 | >128 | 0.125 | >128 | 8 | >128 | 0.125 | <0.06 | 0.5 | 4 | 128 | 0.25 |
| E83 | *E. coli* | 4 | >128 | 2 | 0.25 | >128 | 4 | 32 | 0.125 | <0.06 | 0.5 | 8 | 128 | 1 |
| E84 | *E. coli* | 4 | >128 | 0.5 | 0.25 | >128 | 4 | 64 | 0.125 | <0.06 | 0.5 | 2 | 128 | 0.5 |
| E85 | *E. coli* | 4 | >128 | 2 | 0.25 | >128 | 16 | 64 | <0.06 | <0.06 | 0.5 | 4 | 128 | 0.5 |
| E86 | *E. coli* | 4 | >128 | 2 | 0.25 | >128 | 16 | 64 | <0.06 | <0.06 | 0.5 | 4 | 128 | 0.5 |
| E87 | *E. coli* | 2 | >128 | 128 | 0.125 | >128 | 8 | 2 | <0.06 | <0.06 | 0.25 | 4 | 64 | 0.125 |
| E91 | *E. coli* | 4 | >128 | 16 | >128 | >128 | 64 | 2 | 0.25 | <0.06 | >64 | 64 | 128 | 4 |
| E92 | *E. coli* | 2 | >128 | 16 | 0.25 | >128 | >128 | 128 | 0.125 | <0.06 | 1 | 4 | 128 | 1 |
| E94 | *E. coli* | 4 | >128 | 64 | 0.125 | >128 | 8 | 64 | 0.125 | <0.06 | 1 | 8 | >128 | 0.5 |
| E95 | *E. coli* | 2 | >128 | 16 | >128 | >128 | 8 | 64 | 0.25 | <0.06 | >128 | 8 | 128 | 4 |
| E100 | *E. coli* | 4 | >128 | 128 | 0.25 | >128 | 8 | 64 | 0.125 | <0.06 | 0.5 | 8 | >128 | 0.5 |
| K33 | *K. pneumoniae* | 1 | >128 | 1 | 0.25 | >128 | 2 | 32 | 0.125 | <0.06 | 1 | 8 | 128 | 1 |
| K34 | *K. pneumoniae* | 1 | >128 | 1 | 0.25 | >128 | 128 | 1 | 0.125 | <0.06 | 0.5 | 16 | >128 | 1 |
| K39 | *K. pneumoniae* | 1 | >128 | 2 | 0.25 | >128 | 4 | 64 | 0.25 | <0.06 | 0.25 | 8 | 128 | 1 |
| K40 | *K. pneumoniae* | 2 | >128 | 2 | 0.25 | >128 | 4 | 32 | 0.25 | <0.06 | 0.25 | 8 | >128 | 1 |
| K41 | *K. pneumoniae* | 1 | >128 | 16 | 0.25 | >128 | 16 | 1 | 0.125 | <0.06 | 0.25 | 16 | 128 | 4 |
| K42 | *K. pneumoniae* | 8 | >128 | >128 | 0.25 | >128 | 8 | 128 | 0.125 | <0.06 | 0.25 | 8 | 128 | 2 |
| K62 | *K. pneumoniae* | >128 | >128 | >128 | 0.25 | >128 | 42 | >128 | 0.125 | <0.06 | 0.5 | 16 | 128 | 1 |
| K63 | *K. pneumoniae* | >128 | >128 | >128 | 32 | >128 | 32 | >128 | <0.06 | <0.06 | 32 | 16 | 128 | 1 |
| K64 | *K. pneumoniae* | >128 | >128 | >128 | 32 | >128 | 32 | >128 | 0.125 | <0.06 | 32 | 16 | 128 | 1 |
| K81 | *K. pneumoniae* | 1 | >128 | 0.5 | 0.25 | >128 | 4 | 64 | 0.125 | <0.06 | 0.5 | 8 | 128 | 2 |
| K82 | *K. pneumoniae* | 1 | >128 | 0.5 | 0.25 | >128 | 4 | 64 | 0.125 | <0.06 | 0.5 | 8 | 128 | 2 |

*AMK, amikacin; CFZ, cefazolin; CIP; ciprofloxacin; CST, colistin; CXM, cefuroxime; FOX, cefoxitin; GEN, gentamicin; IMP, imipenem; MEM, meropenem; PMB, polymyxin B; PTZ, piperacillin-tazobactam; TET, tetracycline; TGC, tigecycline; ESBL, extended-spectrum β-lactamase; Neg, negative; Pos, positive. †The MICs were interpreted according to the CLSI guidelines, except for tigecycline, colistin and polymyxin B, which interpretation were performed according to the EUCAST guidelines.
