# Supplementary material for: Occurrence and Genomic Characterization of ESBL-Producing, MCR-1-Harboring Escherichia coli in Farming Soil
Source: Front Microbiol. 2017 Dec 14;8:2510. doi: 10.3389/fmicb.2017.02510 (PMC5735249; doi:10.3389/fmicb.2017.02510)
Supplement: Supplementary file 2 [file Table_2.DOCX]

**Table S2.** Genotypic profile of ESBL-producing *Enterobacteriaceae* isolates from farming soils.

| Isolates, no.† | β-lactamase | | | MLST profile |
| --- | --- | --- | --- | --- |
|  | CTX-M | SHV | TEM |  |
| *E. coli* | | | | |
| 9 | CTX-M-14 | - | - | ST5909 (n=3), ST4977 (n=2), ST746 (n=2), ST877 (n=1), ST204 (n=1) |
| 6 | CTX-M-14 | - | TEM-1 | ST58 (n=1), ST206 (n=1), ST156 (n=1), ST46 (n=1), ST744 (n=1), ST1560 (n=1), |
| 5 | CTX-M-27 | - | TEM-1 | ST10 (n=1), ST6050 (n=2), ST761 (n=1), ST6756 (n=1) |
| 5 | CTX-M-65 | - | - | ST1684 (n=1), ST6246 (n=1), ST5933 (n=1), ST542 (n=1), ST3288 (n=1) |
| 3 | CTX-M-65 | - | TEM-1 | ST3339 (n=1), ST223 (n=1), ST3014 (n=1) |
| 3 | CTX-M-27, CTX-M-55 | - | TEM-1 | ST2060 (n=3) |
| 2 | CTX-M-14, CTX-M-55 | - | TEM-1 | ST2282 (n=1), ST2505 (n=1) |
| 2 | - | - | TEM-1 | ST1286 (n=1), ST2505 (n=1) |
| 1 | CTX-M-55 | - | TEM-1 | ST1196 |
| 1 | CTX-M-15, CTX-M-55 | - | TEM-1 | ST58 |
| 1 | CTX-M-27 | - | - | ST10 |
| 1 | CTX-M-55 | - | - | ST218 |
| 1 | CTX-M-55, CTX-M-65 | - | - | ST218 |
| 1 | CTX-M-65 | SHV-32 | TEM-1 | ST542 |
| 1 | CTX-M-17 | - | TEM-1 | ST2505 |
| *K. pneumoniae* | | | | |
| 3 | CTX-M-27 | SHV-2 | TEM-1 | ST1 |
| 2 | CTX-M-14 | SHV-11 | - | ST37, ST1198 |
| 2 | CTX-M-14 | SHV-32 | - | ST753 |
| 1 | CTX-M-27 | SHV-11 | - | ST661 |
| 1 | CTX-M-11 | - | TEM-1 | ST469 |
| 1 | CTX-M-11 | - | - | ST469 |
| 1 | CTX-M-3 | SHV-2 | TEM-1 | New ST |
