# Supplementary material for: Occurrence and Genomic Characterization of ESBL-Producing, MCR-1-Harboring Escherichia coli in Farming Soil
Source: Front Microbiol. 2017 Dec 14;8:2510. doi: 10.3389/fmicb.2017.02510 (PMC5735249; doi:10.3389/fmicb.2017.02510)
Supplement: Supplementary file 3 [file Table_3.DOCX]

| **Table S3.** Antimicrobial susceptibility profiles of MCR-1-producing *E. coli* and the corresponding transconjugants^*^. | | | | | | | | | | | |
| --- | --- | --- | --- | --- | --- | --- | --- | --- | --- | --- | --- |
| Isolates ID | MIC for antimicrobial drugs tested, μg/mL^†^ | | | | | | | | | | |
|  | AMK | CFZ | CIP | CST | FOX | GEN | IMP | MEM | PMB | PTZ | TGC |
| E11 | 4 | >128 | 64 | 16 | 8 | 128 | 0.125 | <0.06 | 16 | 4 | 0.5 |
| E11co | 4 | >128 | 64 | 16 | 8 | 128 | 0.25 | <0.06 | 16 | 8 | 0.5 |
| E24 | 2 | >128 | 2 | 4 | 4 | 64 | 0.125 | <0.06 | 4 | 2 | 0.25 |
| E24co | 2 | >128 | 8 | 8 | 8 | 64 | 0.125 | <0.06 | 8 | 2 | 0.25 |
| *E. coli* J53 | 0.5 | 4 | 0.25 | 0.5 | 2 | 0.5 | 0.125 | <0.06 | 0.5 | 2 | 0.25 |
| *AMK, amikacin; CFZ, cefazolin; CIP; ciprofloxacin; CST, colistin; FOX, cefoxitin; GEN, gentamicin; IMP, imipenem; MEM, meropenem; PMB, polymyxin B; PTZ, piperacillin-tazobactam; TGC, tigecycline; ESBL, extended-spectrum β-lactamase; Neg, negative; Pos, positive. †The MICs were interpreted according to the CLSI guidelines, except for tigecycline, colistin and polymyxin B, which interpretation were performed according to the EUCAST guidelines. | | | | | | | | | | | |
|  |  |  |  |  |  |  |  |  |  |  |  |
|  |  |  |  |  |  |  |  |  |  |  |  |
|  |  |  |  |  |  |  |  |  |  |  |  |
