# Supplementary material for: Occurrence and Genomic Characterization of ESBL-Producing, MCR-1-Harboring Escherichia coli in Farming Soil
Source: Front Microbiol. 2017 Dec 14;8:2510. doi: 10.3389/fmicb.2017.02510 (PMC5735249; doi:10.3389/fmicb.2017.02510)
Supplement: Supplementary file 4 [file Table_4.DOCX]

| **Table S4.** Genome features of six MCR-1-producing isolates | | | | | | |
| --- | --- | --- | --- | --- | --- | --- |
|  | E4 | E11 | E24 | E38 | E43 | E47 |
| Raw Data (G)^a^ | 1.425 | 1.778 | 1.299 | 1.523 | 1.258 | 1.809 |
| Clean PE (G) | 1.275 | 1.566 | 0.952 | 0.964 | 0.811 | 1.429 |
| Ratio (%) | 89.497 | 88.089 | 73.233 | 63.282 | 63.451 | 79.030 |
| Size (bp) | 4724978 | 4,908,747 | 5072665 | 5050390 | 4828038 | 4885816 |
| Contings number | 109 | 179 | 124 | 116 | 119 | 113 |
| Genes | 4440 | 4,744 | 4833 | 4891 | 4564 | 4628 |
| CDS | 4360 | 4,657 | 4752 | 4804 | 4484 | 4549 |
| tRNA genes | 79 | 86 | 80 | 86 | 79 | 78 |
| rRNA genes | 0 | 0 | 0 | 0 | 0 | 0 |
| tmRNA genes | 1 | 1 | 1 | 1 | 1 | 1 |
| ^a^Raw, paired-end reads were merged after filtering adaptor sequences and removing low-quality reads, ambiguous nucleotides to generate clean sequences | | | | | | |
