# Supplementary figures and images for: Occurrence and Genomic Characterization of ESBL-Producing, MCR-1-Harboring Escherichia coli in Farming Soil
Source: Front Microbiol. 2017 Dec 14;8:2510. doi: 10.3389/fmicb.2017.02510 (PMC5735249; doi:10.3389/fmicb.2017.02510)

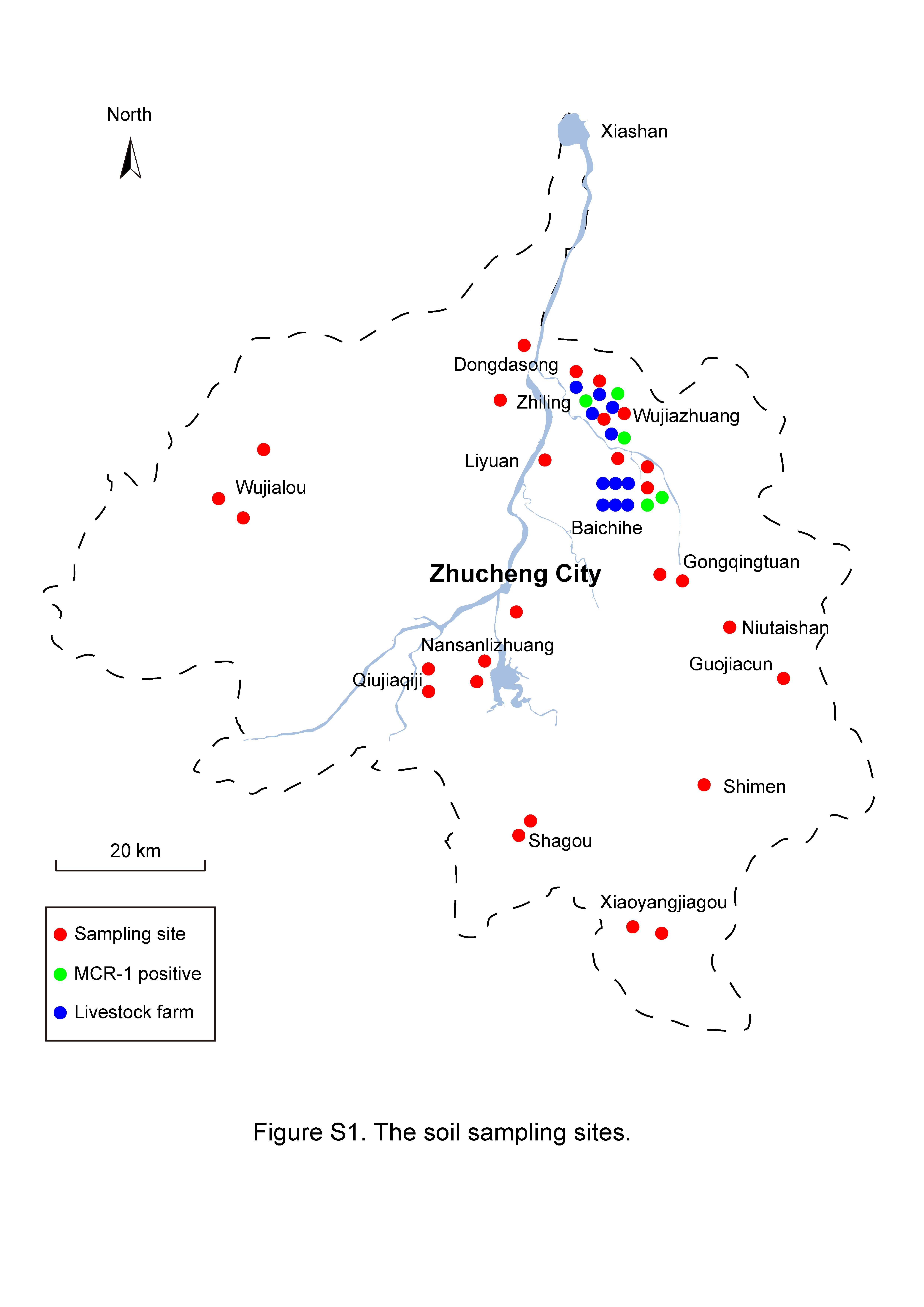

Supplement: Supplementary file 5 [file Image_1.TIF]

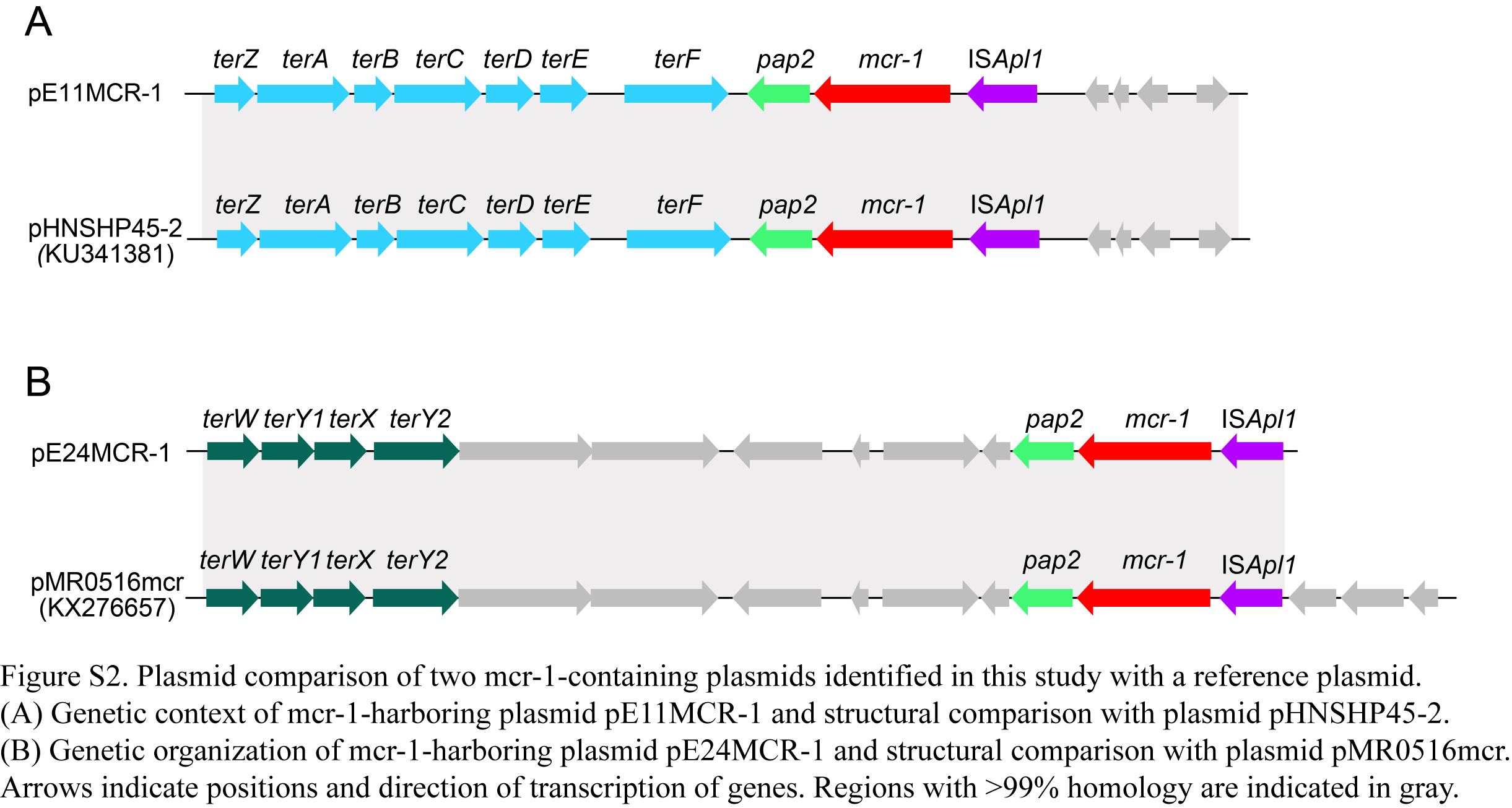

Supplement: Supplementary file 6 [file Image_2.TIF]

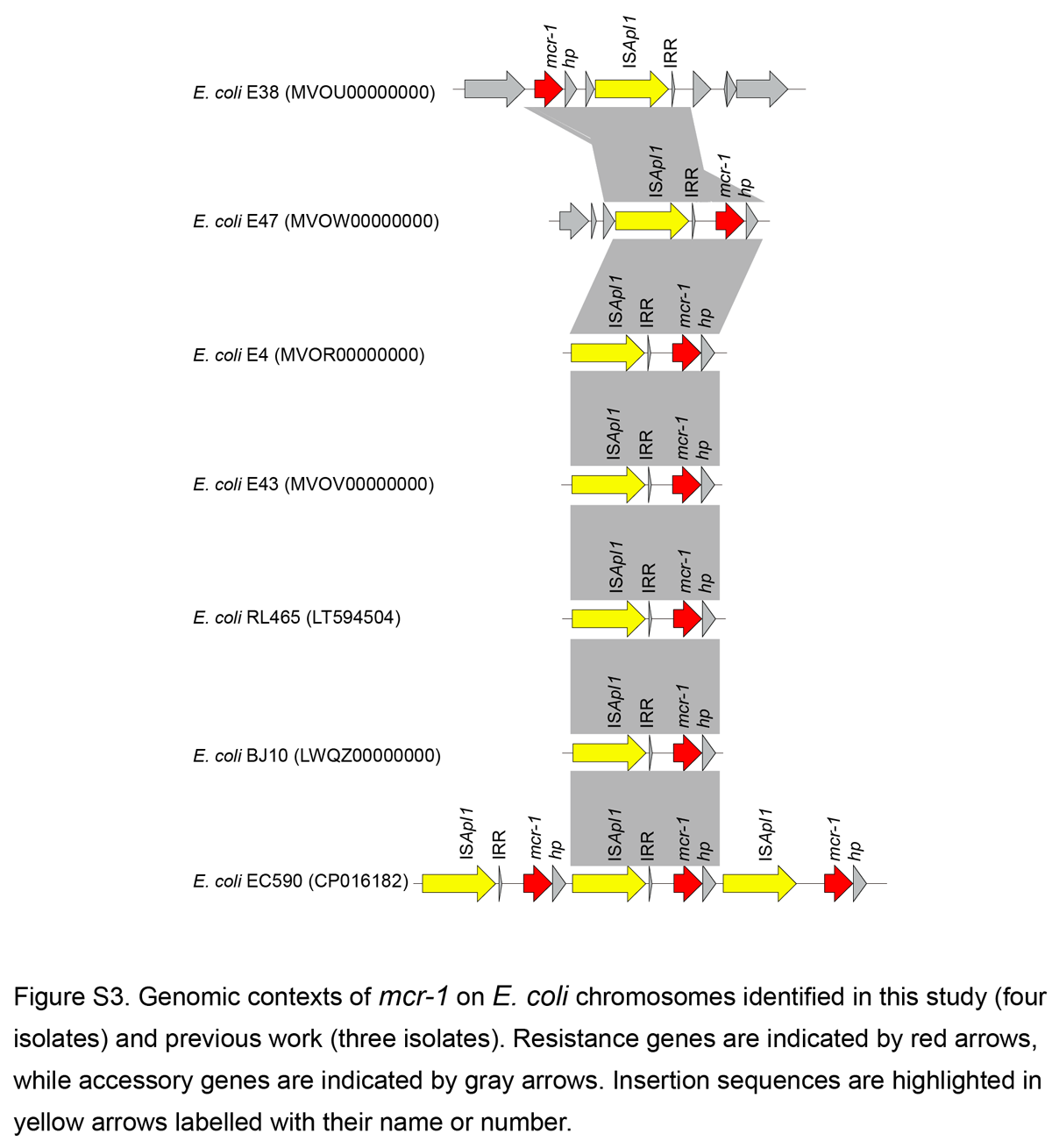

Supplement: Supplementary file 7 [file Image_3.TIF]
